# Origin of sulfate in post-snowball-Earth oceans: river inputs vs. shelf-derived H_2_S

**DOI:** 10.1093/nsr/nwae380

**Published:** 2024-10-25

**Authors:** Huiming Bao, Yongbo Peng, Xiaobin Cao

**Affiliations:** International Center for Isotope Effects Research, Nanjing University, China; Frontiers Science Center for Critical Earth Material Cycling, State Key Laboratory for Mineral Deposits Research, School of Earth Sciences and Engineering, Nanjing University, China; International Center for Isotope Effects Research, Nanjing University, China; Frontiers Science Center for Critical Earth Material Cycling, State Key Laboratory for Mineral Deposits Research, School of Earth Sciences and Engineering, Nanjing University, China; International Center for Isotope Effects Research, Nanjing University, China; Frontiers Science Center for Critical Earth Material Cycling, State Key Laboratory for Mineral Deposits Research, School of Earth Sciences and Engineering, Nanjing University, China

## Abstract

A synthesis of global barite sulfate isotope data from approximately 635 million years ago, at the end of a global glaciation, undermines the hypothesis that river sulfate was the primary carrier of the distinctive 17O-depleted atmospheric O2 signature of the time. Instead, an aqueous H2S oxidation model on the shelf emerges as a compelling alternative, though it demands extensive validation across multiple fronts by the scientific community.

In the earliest Ediacaran, ∼635 million years ago, Marinoan glaciation swiftly ended. Subsequent seawater sulfate recorded a period of pronounced mass-anomalous ^17^O depletion in atmospheric O_2_—a signature that is likely indicative of an exceptionally high CO_2_ concentration at that time [1]. The ^17^O anomaly or Δ′^17^O value of atmospheric O_2_ could have reached approximately −30‰, in contrast to today's value of around −0.5‰ [2] (here, Δ′^17^O ≡ δ′^17^O – 0.5305 × δ′^18^O, where δ′_X_ ≡ ln(R_x_/R_x__–__ref_); R_x_ is the mole ratio of ^18^O to ^16^O or ^17^O to ^16^O; and ‘ref’ refers to VSMOW, which is an international reference material). Basal Ediacaran barite crystal fans have been found on at least five Neoproterozoic paleocontinents and all recorded the ^17^O-depletion event [1,3]. Hoffman *et al.* [4] proposed that these ‘sea-floor barite fans’ precipitated from seawater when sulfidic, Ba-rich deep-water upwelled to the shallow oxic zones. Shields *et al.* [5] also invoked that Ba-rich fluids came into contact with sulfate-rich seawater in explaining the barite fans. While both models underscore the deep-water origin of Ba^2+^, neither elucidates the oxidation pathway that is responsible for the distinct atmospheric O_2_ signature that is observed in the barite sulfate. Based on data from South China, Zhou *et al.* [6] and Peng *et al.* [7] propose a river model in which, despite the fact that Ba^2+^ originated from deep-water sources, sulfate was chiefly supplied by rivers, with a portion derived from the oxidative weathering of land-based sulfide minerals.

Given the accumulation of global sulfur and triple oxygen isotope data for basal Ediacaran barite over recent years, the time is now apt to evaluate the river model. As river sulfate carries the sulfur and most water oxygen isotope signatures of a particular drainage basin, which can be quite heterogeneous throughout the world, the river model predicts similarly heterogeneous δ^34^S and δ^18^O values for the basal Ediacaran barite of different geographic locations.

Globally, we identified only six major rivers with published data on both sulfate δ^34^S and δ^18^O values near their mouths, prior to the influence of seawater (Table S1). For example, the average sulfate δ^34^S and δ^18^O values are –2.7‰ and 3.4‰ for the Mississippi River [8], ∼7‰ and ∼7‰ for the Yellow River [9] and 4.8‰ and –5.2‰ for the Mackenzie River [10], respectively. The arithmetic means of the six paired δ^34^S and δ^18^O values are 4.8‰ ± 4.9‰ and 4.1‰ ± 5.2‰, respectively, and the δ^34^S and δ^18^O values range from –2.7‰ to 10.4‰ and from –5.2‰ to 10.5‰, respectively. The modern river sulfate Δ′^17^O can be considered near-zero and invariant when compared with its Ediacaran counterpart, owing to the minimal Δ′^17^O value that is present in contemporary atmospheric O_2_. The six rivers are sufficiently large in discharge and diverse in geography to represent modern river sulfate δ^34^S and δ^18^O values and ranges. Meanwhile, the compiled basal Ediacaran barite data (Fig. 1 and Table S2) should represent a series of snapshots of shallow-marine sulfate isotope signatures of the early Ediacaran oceans. Its δ^18^O–Δ′^17^O space displays an outstanding feature: the most ^17^O-depleted barite samples of all sites converge toward approximately –1.3‰ in the Δ′^17^O value when their respective δ^34^S and δ^18^O values cluster at around ∼20‰ and ∼17‰. This feature poses a critical challenge to the river model. If river sulfate were indeed the primary source of the ^17^O-depleted barite, then not only are the ranges of the δ^34^S and δ^18^O values too large, but also the average δ^34^S and δ^18^O values of modern river sulfate, at 4.8‰ and 4.1‰, respectively, are markedly too low in comparison with those of basal Ediacaran barite, thus undermining the validity of the river model.

**Figure 1.**
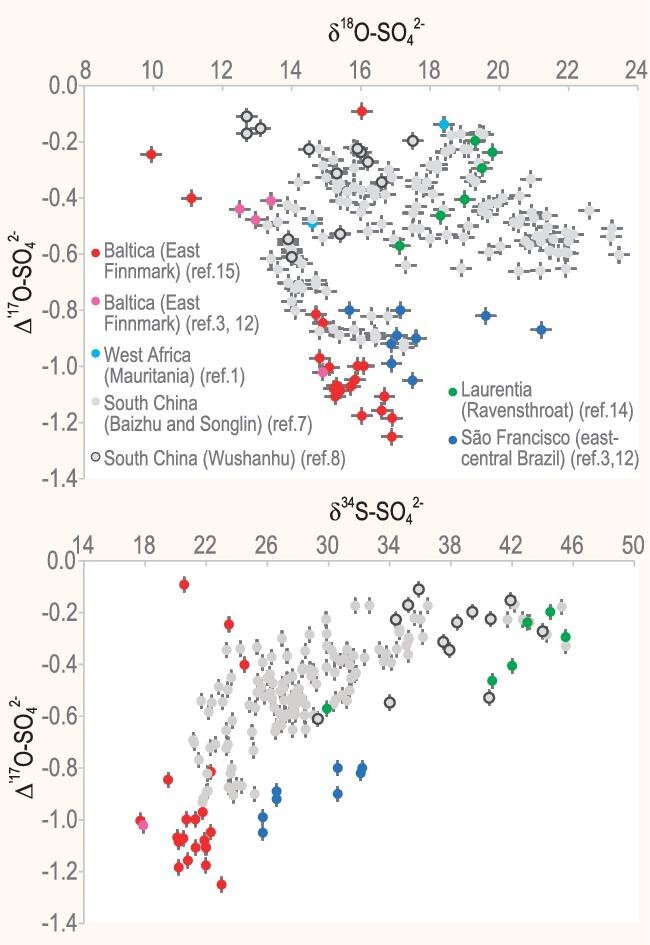
Basal Ediacaran barite δ^18^O–Δ′^17^O plot (upper panel) and δ^34^S–Δ′^17^O plot (lower panel). Data source: [1,3,7,12–15]. All Δ′^17^O values were reported by using the exponent of 0.5305 (Table S2).

The substantial discrepancies indicate that the basal Ediacaran ^17^O-depleted barite sulfate could not have been predominantly derived from the oxidative weathering of land-based sulfide minerals. A possibly more negative seawater δ^18^O value in the Ediacaran than today can only exacerbate the discrepancy and post-depositional alteration must be minimal, as the ^17^O anomalies would have been erased if there were any incorporation of a late fluid signature. Here, we propose that H_2_S (or HS^−^) oxidation at the oxic-sulfidic redoxcline on the shelf, not the river input, was the dominant source of the ^17^O-depleted SO_4_^2−^. In the early Ediacaran, the overall low seawater SO_4_²^–^ concentration often led to its complete reduction to H_2_S, which accumulated in the water column for as long as the organic flux had exhausted O_2_ and the supply of Fe(II) was limited. The fluctuating redoxcline brought SO_4_^2−^-free, Ba^2+^-bearing water to meet the oxic water. The H_2_S/HS^–^ encountered dissolved O_2_, which resulted in their oxidation into SO_4_^2^^–^ that was subsequently reacted with Ba^2^^+^ to precipitate as barite.

A first prediction of our H_2_S model is that the δ^18^O of SO_4_^2–^ is *∼*17‰–22‰ higher than that of H_2_O, assuming that the early Ediacaran seawater δ^18^O is at 0 to −5‰. Today, the oxygen isotope systematics for H_2_S oxidation in aerobic water are poorly known. Laboratory experiments and natural observations indicate that a δ^18^O difference of this magnitude, as predicted by the H_2_S model, is feasible but lacks detailed verification (e.g. [11]), necessitating experimental validation of aqueous aerobic H_2_S oxidation. A second prediction is that the dissolved O_2_ at redoxcline had an air O_2_ signal and was incorporated into the product SO_4_^2‒^ during H_2_S oxidation. To date, no experiment has quantified the proportion of dissolved O_2_ in the SO_4_^2‒^ that was produced this way, being biotic or abiotic. A third prediction is a global presence of SO_4_^2–^-free sulfidic water on the early Ediacaran shelf. Such a water mass is not found in any contemporary oceanic environments, including well-known permanently anoxic deep waters such as those in the Black Sea or the Cariaco Trench. Currently, all sulfidic or euxinic water masses exhibit relatively high sulfate concentrations. A global presence of SO_4_^2–^-free sulfidic water would predict a homogeneous Ba isotope composition for most basal Ediacaran barite crystal fans on different paleocontinents.

The additional constraints that are provided by the high-dimensional isotope signature Δ′^17^O has highlighted our deficiencies in understanding on two distinct levels. At the molecular level, there are unresolved biotic or abiotic pathways that incorporate dissolved atmospheric O_2_ into sulfate during sulfide oxidation. At the paleo-ocean redox history level, we are prompted to consider a scenario in which H_2_S-rich, SO_4_^2−^-free water masses could have been globally prevalent during the early Ediacaran or possibly throughout much of the late Proterozoic. Further examination of the proposed hypothesis offers a distinct advantage: it may uncover an uncharted and intriguing record of atmospheric O_2_ signatures from the distant past.

## Supplementary Material

nwae380_Supplemental_File

## Data Availability

All data used in this study have been tabulated and are accessible in the Supplementary data. All data have been previously published.
