# Supplementary material for: Origin of sulfate in post-snowball-Earth oceans: river inputs vs. shelf-derived H2S
Source: Natl Sci Rev. 2024 Oct 25;11(11):nwae380. doi: 10.1093/nsr/nwae380 (PMC11565228; doi:10.1093/nsr/nwae380)
Supplement: nwae380_Supplemental_File [file nwae380_supplemental_file.pdf]

Origin of Sulfate in Post-Snowball-Earth Oceans: River Inputs vs. Shelf-Derived H_2S

Huiming Bao[1,2*], Yongbo Peng[1,2], Xiaobin Cao[1,2]

[1]*International Center for Isotope Effects Research, Nanjing University, Nanjing 210023, China*
[2]*School of Earth Sciences and Engineering, Nanjing University, Nanjing 210023, China*
*Corresponding author, bao@nju.edu.cn

Supplementary Data Table 1. *Paired river sulfate $\delta^{34}S$ and $\delta^{18}O$ values for major rivers that flow into the oceans. Only data near the mouth of a river but not influenced by seawater were compiled. Most of the mean values were calculated based on published raw data.*

Ocean-bound rivers	$\delta^{34}S$ ‰	$\delta^{18}O$ ‰	Notes	References
Mississippi River, U.S.A.	-2.7	3.4	4-year average at Baton Rouge, ~100 km from the mouth	[1]
Yellow River, China	~7	~7	Site M23, ~200 km from the mouth	[2]
Yangtze River, China	10.4	5.1	Wuhan, ~800 km from the mouth	[3]
Llobregat River, Spain	8.4	10.5	Site 49, ~30 km from the mouth	[4]
Indus River, Pakistan	0.8	4.2	Site 25, ~100 km from the mouth	[5]
Mackenzie River, Canada	4.8	-5.2	Site 2, ~150 km from the mouth	[6]
Arithmetic mean	**4.8**	**4.1**		
Standard deviation (1σ)	4.9	5.2		

References for Supplementary Data Table 1

1. Killingsworth BA, Bao HM, Kohl IE. Assessing Pyrite-Derived Sulfate in the Mississippi River with Four Years of Sulfur and Triple-Oxygen Isotope Data. *Environmental Science & Technology*. 2018; **52**(11): 6126-6136. doi: 10.1021/acs.est.7b05792
2. Zhang D, Zhao Z-Q, Peng Y *et al.* Sulfur cycling in the Yellow River and the sulfate flux to the ocean. *Chemical Geology*. 2020; **534**: 119451. doi: https://doi.org/10.1016/j.chemgeo.2019.119451
3. Li X, Gan Y, Zhou A *et al.* Relationship between water discharge and sulfate sources of the Yangtze River inferred from seasonal variations of sulfur and oxygen isotopic compositions. *Journal of Geochemical Exploration*. 2015; **153**: 30-39.
4. Otero N, Soler A, Canals A. Controls of $d^{34}S$ and $d^{18}O$ in dissolved sulphate: Learning from a detailed survey in the Llobregat River (Spain). *Applied Geochemistry*. 2008; **23**(5): 1166-1185. doi: 10.1016/j.apgeochem.2007.11.009
5. Karim A, Veizer J. Weathering processes in the Indus River Basin: implications from riverine carbon, sulfur, oxygen, and strontium isotopes. *Chemical Geology*. 2000; **170**(1-4): 153-177.
6. Calmels D, Gaillardet J, Brenot A *et al.* Sustained sulfide oxidation by physical erosion processes in the Mackenzie River basin: Climatic perspectives. *Geology*. 2007; **35**(11): 1003-1006. doi: 10.1130/g24132a.1

Supplementary Data Table 2

Basal Ediacaran barite sulfur and triple oxygen isotope data

Sample name	$\Delta'^{17}O$-0.5305 (recalculated)	$\delta^{18}O$	$\delta^{34}S$
	**n.a.: not available due to small sample sizes*		
**Baltica (East Finnmark) (ref.15)**			
EF-64/2012 3/3-1	-1.07	15.3	20.1
EF-64/2012 3/3-2	-1.09	15.4	20.2
RB14-1	-1.16	16.6	20.8
RB14-2	-0.85	14.9	19.5
RB14-15	-1.18	16.9	20.2
RB14-16	-1.00	15.9	20.7
RB14-17	-1.07	15.7	20.5
RB14-18	-0.40	11.1	24.5
RB14-20	-0.81	14.7	22.3
EF-64-2012-1-2	-1.11	16.7	22.0
EF-64-2012-1-2	-1.11	16.7	22.0
EF-64-2012-1-3	-1.18	16.0	22.0
EF-64-2012-2-2019	-1.00	16.1	21.3
EF-64-2012-3-2019	-1.05	15.8	22.3
EF-64-2012-3-2019-2	-0.97	14.8	21.8
RB14-1-2019	-1.08	15.2	21.9
RB-14-15-1-1	-0.09	16.0	20.6
RB14-15-1-2	-1.25	16.9	23.0
RB14-17-2019	-1.11	15.3	21.3
RB14-18-2019	-0.25	9.9	23.5
RB14-2-2	-1.00	15.1	17.7
RB14-2-3	n.a.	15.3	20.9
RB14-2-4	n.a.	15.2	20.8
RB14-15-2-1	n.a.	15.8	21.5
RB14-2-1	n.a.	15.3	20.6
**Baltica (East Finnmark) (ref.3, 12)**			
PCFN-1	-0.48	13.0	n.a.
PCFN-2	-0.44	12.5	n.a.
PCFN-4	-0.41	13.4	n.a.
PCFN-5	-1.02	14.9	17.880
**West Africa (Mauritania) (ref.1)**			
C21	-0.14	18.4	n.a.
C24	-0.49	14.6	n.a.
**South China (Baizhu and Songlin) (ref.7)**			
Jinhe-08-1-1	-0.66	13.6	23.3
Jinhe-08-1-1-1	-0.72	14.3	22.3
Jinhe-08-1-1-2	-0.62	13.4	23.7

Jinhe-08-1-1-R	-0.89	16.4	22.0
Jinhe-08-1-2-R	-0.93	17.2	21.7
Jinhe-08-1-3-R	-0.92	17.2	21.8
Jinhe-08-1-4-R	-0.82	16.8	22.1
Jinhe-08-1-5-R	-0.89	15.4	22.1
Jinhe-08-2-1	-0.44	14.1	26.2
Jinhe-08-2-B	-0.49	16.6	27.1
Jinhe-08-3-B	-0.37	16.0	26.3
Jinhe-3t	-0.73	15.0	25.1
Jinhe-3t-1-1	-0.72	14.1	23.3
Jinhe-3t-1-1-1	-0.87	15.2	24.0
Jinhe-3t-1-2	-0.71	14.2	23.4
Jinhe-3t-2	-0.71	14.7	22.6
Jinhe-3t-3	-0.54	14.9	21.7
Jinhe-3t-4	-0.50	13.3	23.4
NC-11	-0.52	16.2	30.9
NC-11-1	-0.38	15.6	31.0
NC-11-2	-0.23	14.8	29.8
OB-4-1-1	-0.57	20.0	27.8
OB-4-1-2	-0.49	20.6	28.1
OB-4-1-3	-0.55	21.6	28.0
OB-4-1-4	-0.55	22.0	27.6
OB-4-1-5	-0.59	21.5	27.5
ZB-07-24	-0.51	17.5	25.3
ZB-07-25	-0.57	13.9	26.4
ZB-07-26	-0.34	19.3	31.2
SL-1	-0.41	15.4	29.9
SL-1-1	-0.35	15.4	28.5
SL-1-2	-0.29	15.8	36.2
SL-1-2-1	-0.33	16.9	35.1
SL-1-2-2	-0.39	16.4	34.1
SL-1-2-3	-0.26	15.2	34.6
SL-1-3	-0.33	15.0	29.9
SL-2	-0.34	17.9	30.2
SL-2-1	-0.55	18.0	23.1
SL-2-1-R	-0.54	19.0	31.2
SL-2-2	-0.51	17.7	26.2
SL-2-2-R	-0.45	17.5	31.5
SL-2-3	-0.39	16.7	33.6
SL-2-3-R	-0.44	18.4	31.7
SL-2-4-R	-0.37	17.8	31.6
SL-2-5-R	-0.39	18.1	30.8
SL-2-6-R	-0.43	19.5	31.8
SL-3	-0.49	13.6	22.8
SL-3-1	-0.58	14.0	22.2
SL-3-2	-0.56	13.9	24.8
SL-4	-0.31	15.3	35.2

SL-4-1-1	-0.82	16.3	23.6
SL-4-1-2	-0.36	15.8	35.2
SL-4-2-1	-0.34	15.3	33.3
SL-4-2-2	-0.22	15.8	35.6
SL-5	-0.37	15.3	27.3
SL-6-1	-0.41	15.6	28.3
SL-6-1-2-1	-0.42	16.1	28.9
SL-6-1-2-2	-0.28	14.9	28.8
SL-6-2	-0.37	16.3	30.4
ZB-07-14	-0.34	17.6	24.2
ZB-07-14-2-1	-0.55	17.0	22.4
ZB-07-14-2-2	-0.42	19.9	28.8
ZB-07-14-2-3	-0.34	19.3	34.5
ZB-07-14-2-4	-0.17	19.5	36.5
ZB-07-14-2-5	-0.23	19.0	36.0
ZB-07-14-2-6	-0.27	19.4	34.6
ZB-07-14-2-7	-0.17	18.9	32.7
ZB-07-14-2-8	-0.17	19.5	31.7
ZB-07-17	-0.43	17.6	30.4
ZB-07-18-1	-0.53	21.3	28.2
ZB-07-18-1-1-1	-0.50	19.1	31.4
ZB-07-18-1-1-10	-0.60	23.5	27.1
ZB-07-18-1-1-11	-0.54	23.2	27.1
ZB-07-18-1-1-2	-0.59	21.9	27.2
ZB-07-18-1-1-3	-0.65	21.9	27.6
ZB-07-18-1-1-4	-0.60	22.0	28.4
ZB-07-18-1-1-5	-0.60	21.7	29.0
ZB-07-18-1-1-6	-0.45	22.6	28.9
ZB-07-18-1-1-7	-0.56	21.2	28.5
ZB-07-18-1-1-8	-0.51	23.3	27.9
ZB-07-18-1-1-9	-0.53	22.1	27.6
ZB-07-18-1-2-1	-0.53	18.5	26.3
ZB-07-18-1-2-2	-0.66	20.6	25.0
ZB-07-18-1-2-3	-0.46	21.4	25.4
ZB-07-18-1-2-4	-0.39	20.6	25.7
ZB-07-18-1-2-5	-0.57	20.9	25.2
ZB-07-18-1-2-6	-0.33	20.9	25.3
ZB-07-18-2	-0.59	19.7	27.4
ZB-07-18-2-1-1	-0.66	21.2	26.5
ZB-07-18-2-1-2	-0.47	21.0	26.3
ZB-07-18-2-1-3	-0.59	21.4	26.8
ZB-07-18-2-1-4	-0.50	21.4	26.8
ZB-07-18-2-1-5	-0.55	20.7	30.3
ZB-07-18-2-1-6	-0.54	21.1	30.5
ZB-07-18-2-2-1	-0.46	19.7	27.3
ZB-07-18-2-2-2	-0.52	21.9	25.8
ZB-07-18-2-2-3	-0.45	20.9	26.0

ZB-07-18-2-2-4	-0.49	20.4	25.6
ZB-07-18-3	-0.65	18.9	28.5
ZB-07-20	-0.23	19.1	41.7
ZB-07-20-1-1	-0.18	18.6	45.3
ZB-07-20-1-2	-0.28	18.0	44.3
ZB-07-20-1-3	-0.23	18.6	42.7
ZB-07-20-2-1	-0.33	19.2	45.5
ZB-07-20-2-2	-0.24	18.4	43.2
ZB-07-20-2-3	-0.17	19.4	42.2
ZB-07-22	-0.40	20.1	31.4
ZB-07-24-1-1	-0.64	17.3	26.6
ZB-07-24-1-2	-0.45	16.0	23.6
ZB-07-24-2-1	-0.34	14.2	23.3
ZB-07-24-2-2	-0.87	15.8	24.3
ZB-07-24-3-1	-0.91	16.0	23.8
ZB-07-24-3-2	-0.88	16.4	23.6
ZB-07-24-3-3	-0.90	16.4	25.2
ZB-07-24-3-4	-0.62	14.2	26.8
ZB-07-24-3-5	-0.63	14.3	26.9
ZB-07-24-3-6	-0.59	14.0	27.8
ZB-07-24-3-7	-0.43	13.9	29.0
ZB-07-24-B	-0.48	14.6	27.0
ZB-07-25-1	-0.87	14.8	23.5
ZB-07-25-2	-0.80	14.1	23.7
ZB-07-25-3	-0.77	14	21.5
ZB-07-25-4	-0.70	13.9	21.2
ZB-07-25-b-D	-0.69	14.7	21.1
ZB-07-26-1-1	-0.30	18.2	35.2
ZB-07-26-1-2	-0.30	16.8	27.0
ZB-07-26-1-3	-0.28	18.2	29.9
ZB-07-26-1-4	-0.36	17.6	33.8
ZB-07-26-1-5	-0.37	16.4	32.4
ZB-07-26-2-1	-0.45	19.5	27.6
ZB-07-26-2-2	-0.47	20.6	29.2
ZB-07-26-2-3	-0.42	19.7	28.3
**South China (Wushanhu) (ref.8)**			
ZB11-118-AA	-0.11	12.7	35.9
ZB11-118-E	-0.53	15.4	40.5
ZB11-118-H	-0.61	14.0	29.3
ZB11-120-D	-0.31	15.3	37.5
ZB11-120-B	-0.34	16.6	37.9
ZB11-121-A	-0.55	13.9	34.0
ZB11-123-A	-0.24	16.0	38.4
ZB11-123-B	-0.23	14.5	34.4
ZB11-123-DA	-0.17	12.7	35.2
ZB11-124-B	-0.22	15.9	40.6

ZB11-124-FA	-0.15	13.1	41.9
ZB11-124-L	-0.20	17.5	39.4
ZB11-124-KA	-0.27	16.2	44.0
**Laurentia (Ravensthroat) (ref.14)**			
2-2	-0.57	17.1	29.9
4-1	-0.46	18.3	40.7
4-2	-0.41	19.0	42.0
3-1	-0.29	19.5	45.5
3-2	-0.24	19.8	43.0
3-3	-0.20	19.3	44.5
**São Francisco (east-central Brazil) (ref.3,12)**			
PCSL-1	-0.87	21.2	n.a.
PCSL-2	-0.80	15.7	30.6
PCSL-3	-0.90	17.6	30.6
PCSL-4	-0.80	17.1	32.2
PCSL-5	-0.82	19.6	32.1
PCSL-6	-1.05	17.5	25.7
PCSL-7	-0.99	16.9	25.7
PCSL-8	-0.89	17.0	26.6
PCSL-9	-0.92	16.9	26.6
